# Innovative endovascular stapling technique using the Aortoseal device in challenging infrarenal aortic neck

**DOI:** 10.1016/j.jvscit.2026.102144

**Published:** 2026-01-10

**Authors:** Emidio Germano, Animesh Rathore, David J. Dexter

**Affiliations:** Macon and Joan Brock Virginia Health Sciences Eastern Virginia Medical School at Old Dominion University, Norfolk, VA

**Keywords:** Aortic aneurysm, Complex aortic aneurysm repair, EndoAnchor

## Abstract

We report the use of the Aortoseal Endostapling system by Endoron in a 63-year-old man with infrarenal abdominal aortic aneurysm with high-risk neck features. Endovascular abdominal aneurysm repair was performed using a Medtronic Endurant IIs endograft with an adjunctive Aortoseal system as part of an international feasibility trial. The procedure was technically successful, with no endoleaks on completion angiography. Follow-up computed tomography angiography at 1 month confirmed a well-deployed AortoSeal system and complete aneurysm exclusion. This case highlights this innovative device and the technical considerations to enhance endograft proximal fixation with the potential to improve long-term outcomes in endovascular aneurysm repair.

Endovascular aneurysm repair (EVAR) has become the preferred treatment for abdominal aortic aneurysms (AAAs), yet approximately 40% to 60% of patients are considered to be outside the instruction for use owing to unfavorable anatomy, most commonly owing to high-risk neck features.^1^ Given the progressive nature of this disease, endoleaks at the proximal fixation zone (type Ia) remain a major deterrent in long-term outcomes after EVAR.^2^^,^^3^ To address this challenge, the Aortoseal Endostapling system (Endoron Medical Holdings) developed a novel device designed to mimic the hand-sewn anastomosis of open aortic repair by simultaneously delivering multiple transmural nitinol staples circumferentially to reinforce the endograft proximal seal zone. This case report demonstrates the application of the Aortoseal as an adjunct to EVAR in a patient with suboptimal neck anatomy. This device was used under an investigational device exemption as part of the early feasibility phase of SEAL (Safety and Feasibility Evaluation of the Aortoseal Endostapling System). This case report was approved by the SEAL trial organizers and our local institutional review board (IRB #25-07-NH-0175). The patient signed informed consent for publication of this case report.

## Case report

**Fig 1:**
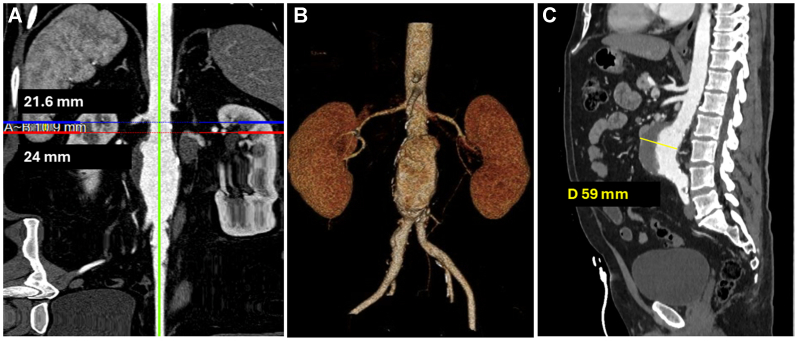
**(A)** Straightened multiplanar reconstruction of the aortic neck, showing proximal (DA) and distal (DB) diameters. A∼B indicates the centerline length of the aortic neck. **(B)** A three-dimensional (3D) reconstruction of the abdominal aorta. **(C)** Coronal views showing the thrombus-laden anterior abdominal wall and an aortic diameter measuring 59 mm.

A 63-year-old man—a former smoker with type 2 diabetes mellitus and coronary artery disease—presented with an incidentally discovered AAA on a screening chest computed tomography (CT) scan for lung cancer. He denied any symptoms of abdominal or back pain. Subsequent CT angiography revealed an infrarenal AAA with maximum diameter of 59 mm. The aortic neck measured 10.9 mm in length and demonstrated a reverse taper from 21.6 to 24.0 mm, and moderate mural thrombus burden (Fig 1), which raised concerns for long-term durability after standard infrarenal EVAR. Suprarenal and infrarenal angulation measured 25° and 14°, respectively. A preoperative nuclear stress test showed reversible mild to moderate inferior and apicolateral wall defects. The patient denied anginal symptoms or dyspnea on exertion. Although he was not at prohibitive risk for open infrarenal aortic repair, the patient elected to undergo endovascular intervention after extensive preoperative discussion.

We proceeded with EVAR using a 28 × 14 × 103 mm Medtronic Endurant endograft with adjunctive Aortoseal endostapling. After endograft deployment, right femoral access was maintained with a Gore 18F Dryseal sheath. The Aortoseal system was advanced to the proximal 5-mm infrarenal seal zone. The delivery catheter was centered using multiple orthogonal views. The nitinol frame was then unsheathed and deployed, with careful attention to stability and correct positioning 5 mm caudal to the most proximal endograft edge. This was followed by the proprietary dual balloon catheter system. It includes a distal stabilizing balloon that was inflated just above the endograft flow divider to provide additional stability.

**Fig 2:**
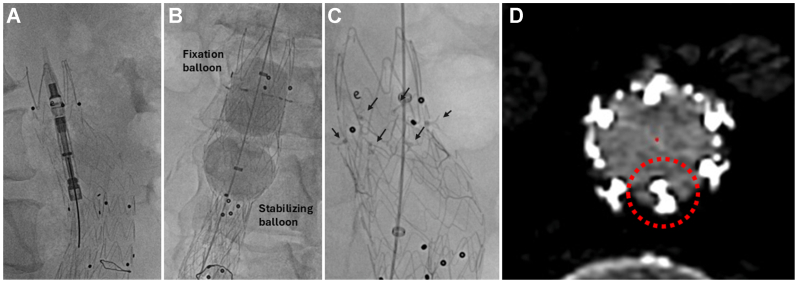
**(A)** Still image demonstrating unsheathing of the Aortoseal staple delivery system. **(B)** Dual-balloon catheter inflated within the endograft, with the stabilizing balloon positioned just above the endograft bifurcation and the fixation balloon within the Aortoseal scaffold. **(C)** Completion angiographic image after endostaple deployment. *Black arrows* demonstrate the location of the endostaples. **(D)** Cone-beam computed tomography (CT) image demonstrating circumferential distribution of endostaples along the nitinol frame. The *red dashed circle* indicates the nondeployed endostaple.

**Fig 3:**
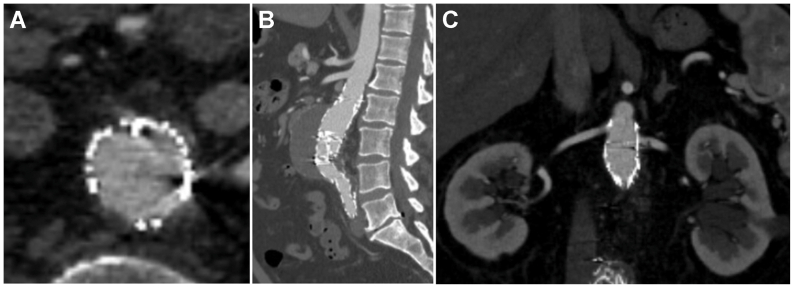
**(A-C)** One-month postoperative computed tomography (CT) angiography demonstrating a well-opposed endograft with no evidence of endoleaks.

Next, the proximally located fixation balloon was inflated with a contrast-saline mixture to a pressure of 2 ATM, while monitoring simultaneous endostaple penetration under fluoroscopy. Completion aortogram demonstrated a good proximal seal with no type I endoleak, and successful penetration of five out of six endostaples, consistent with technical success (Fig 2). Total fluoroscopy time was 29.8 minutes, with a radiation dose of 1246 mGy. The patient had an uneventful postoperative course and was discharged home on postoperative day 1. At the 1-month follow-up, CT angiography (Fig 3) confirmed five out of six deployed endostaples with no endograft migration and no type Ia endoleaks.

## Discussion

Loss of proximal endograft seal is a leading cause of EVAR failure, notably in patients with short or angulated aortic necks.^4^^,^^5^ Conventional EVAR techniques rely on radial force and device-specific active fixation mechanisms that may not prevent loss of wall apposition in challenging anatomy.^6^^,^^7^ Adjunctive aortic fixation devices, such as the Medtronic Heli-FX EndoAnchors (Medtronic), have been created and proven beneficial to prevent type Ia endoleaks.^8^^,^^9^ However, data from ANCHOR (Aneurysm Treatment using the Heli-FX EndoAnchor System Global Registry) showed rates of inaccurate deployment in 30% of anchors and an additional 13% achieving only borderline depth.^10^ Each EndoAnchor is deployed under fluoroscopy using unique orthogonal views.^11^ This multistep process may result in deployment angulation errors and suboptimal results owing to incomplete penetration and asymmetrical deployment across the proximal landing zone.^12^

The Aortoseal system offers key technical advantages aimed at addressing these limitations. It offers a single-stage deployment of six evenly spaced V-shaped nitinol staples deployed simultaneously in a single stage mechanism.^13^ This configuration reinforces the proximal seal zone by delivering transmural aortic fixation while augmenting endograft apposition by the added radial force of the nitinol frame. An additional system component is the dual balloon catheter. The distally located stabilizing balloon mitigates wind-socking effects during proximal fixation balloon inflation. This added stability may reduce shear forces and the risk of aortic wall injury during endostaple deployment compared with traditional aortic occlusion or molding balloons, such as the Coda or Reliant balloons. These technical features may contribute to more consistent outcomes and a more effective proximal seal.

The Aortoseal requires only two orthogonal fluoroscopic views for the deployment of all endostaples, thereby potentially reducing radiation exposure and procedure time. In our case, the total fluoroscopy time of 29.8 minutes was slightly shorter than the average time of 35.3 minutes reported in a subgroup analysis of the ANCHOR registry.^14^ Because this was our first experience using the Aortoseal device, we anticipate shorter procedure times and lower radiation doses with increasing operator experience.

The AortoSeal's endograft-agnostic design supports its use across a broad range of EVAR cases. Currently, this investigational device is indicated for use in cases with an aortic neck length of ≥10 mm. Similar to Heli-FX EndoAnchors, heavy aortic wall calcification, mural thrombus, and neck angulation >75° may interfere with staple penetration and fixation.^13^^,^^15^ Technical success is defined as the full deployment of at least four out of six endostaples. In our case, the nondeployment of one endostaple was likely caused by interference with the endograft stent struts, a limitation that needs to be addressed in future device iterations. Of note, the endostaples are integrated into the nitinol frame, such that any endostaples that do not deploy remain attached to the frame and do not pose a risk of embolization.

As EVAR expands into more anatomically complex cases, adjunctive devices like the Aortoseal provide a promising, low-complexity method to reinforce proximal sealing. This case highlights the device's potential to improve procedural durability and long-term outcomes in EVAR. Prospective studies will be essential to validate its broader clinical applicability and long-term efficacy. As the SEAL trial expands and follow-up data mature, outcomes related to endoleak prevention will be systematically analyzed and reported.

## Conclusions

The Aortoseal offers a safe, low-complexity adjunctive strategy to enhance infrarenal neck seal in EVAR, particularly in patients with hostile neck anatomy. This case supports the feasibility and potential of this novel endovascular stapling system to improve outcomes in EVAR.

## Funding

None.

## Disclosures

A.R. and D.J.D. are consultants for Medtronic.
